# FcGBP was upregulated by HPV infection and correlated to longer survival time of HNSCC patients

**DOI:** 10.18632/oncotarget.21220

**Published:** 2017-09-23

**Authors:** Yating Wang, Yan Liu, Huiqiao Liu, Qingan Zhang, Hongyan Song, Jianliang Tang, Jiangtao Fu, Xiaofei Wang

**Affiliations:** ^1^ Department of Otolaryngology Head and Neck Surgery, Lishui People’s Hospital, The Sixth Affiliated Hospital of Wenzhou Medical University, Lishui, Zhejiang, China; ^2^ Department of Clinical Laboratory, The Central Hospital of Linyi, Yishui, Shandong, China; ^3^ Department of Otolaryngology, Yunhe People’s Hospital, Yunhe, Zhejiang, China; ^4^ Department of Psychiatry, Tongxiang First People’s Hospital, Tongxiang, Zhejiang, China

**Keywords:** FcGBP, HPV, HNSCC, TGF-β, migration

## Abstract

FcGBP was normally found in intestinal and colonic epithelia, gallbladder, cystic duct, bronchus, submandibular gland, cervix uteri and in fluids secreted by these cells in humans, and was down-regulated during colon carcinogenesis. We found FcGBP gene expression was decreased in HNSCC tissues compared to surgical safety border tissues while TGF-β expression level increased in HNSCC tissues, and higher FcGBP expression level was correlated to longer OS time of HNSCC patients. FcGBP expression level was higher in HPV-positive HNSCC tissues compared to HPV-negative HNSCC tissues, while TGF-β expression level was lower in HPV-positive HNSCC tissues. Gene expression level of FcGBP and TGF-β was negatively correlated in HNSCC tissues. FcGBP expression level increased after HPV E6 overexpression in HPV-negative HNSCC cells, and TGF-β could inhibit the up-regulation of FcGBP after HPV E6 or FcGBP overexpression in HPV-negative HNSCC cells. The migration capability was inhibited after FcGBP overexpression, and TGF-β could counteract the inhibition of migration caused by FcGBP overexpression. FcGBP gene expression level was correlated to the expression levels of EMT markers. In conclusion, FCGBP expression was upregulated by HPV infection while inhibited by TGF-β, and was correlated to the prognosis of HNSCC patients.

## INTRODUCTION

Head and neck squamous cell carcinoma (HNSCC) accounts for about 6% of all human cancers, with about 650,000 new cases reported and 350,000 HNSCC-related deaths per year worldwide [1]. Tobacco, alcohol consumption and human papillomavirus (HPV) infection are the main etiological factors [2–4]. The biological behavior, prognosis and genetic landscape are significantly different between HPV-positive and HPV-negative HNSCC patients, and many studies have found that HPV-positive HNSCC patients have better prognosis, with increased survival time and less chance of recurrence [5–8].

FcGBP protein was originally isolated from the small intestine that bound the Fc portion of immunoglobulin [9–12]. FcGBP is a ∼300 kDa protein with multiple Von Wille-brand factors and mucin-like repeats [11]. FcGBP has been reported to be downregulated in ulcerative colitis, which is a chronic inflammatory disease predisposing to colorectal cancer (CRC) [13, 14]. FcGBP may play an important role in anti-inflammation and cell protection in epithelium cells [15]. The mutation of FcGBP has been previously identified in CRC [16]. Furthermore, FcGBP was significantly enriched in the function of cell adhesion. Metastasis is facilitated by the cell-cell interactions between the endothelium and tumor cells in targeted organs. Cell adhesion occurring in vasculature of specific organs is an essential step in cancer metastasis [17].

In this paper, we found FcGBP gene expression was downregulated in HNSCC tissues and correlated with overall survival time in HNSCC patients. Furthermore, we found FcGBP gene expression level was higher in HPV-positive than HPV-negative HNSCC tissues while TGF-β gene expression level was lower in HPV-positive HNSCC tissues, and FcGBP gene expression was inhibited by TGF-β. This network played an important role in HPV related tumorigenesis and HNSCC progression.

## RESULTS

### FcGBP gene expression was down regulated in HNSCC and negative correlated to TGF-β gene expression

**Figure 1:**
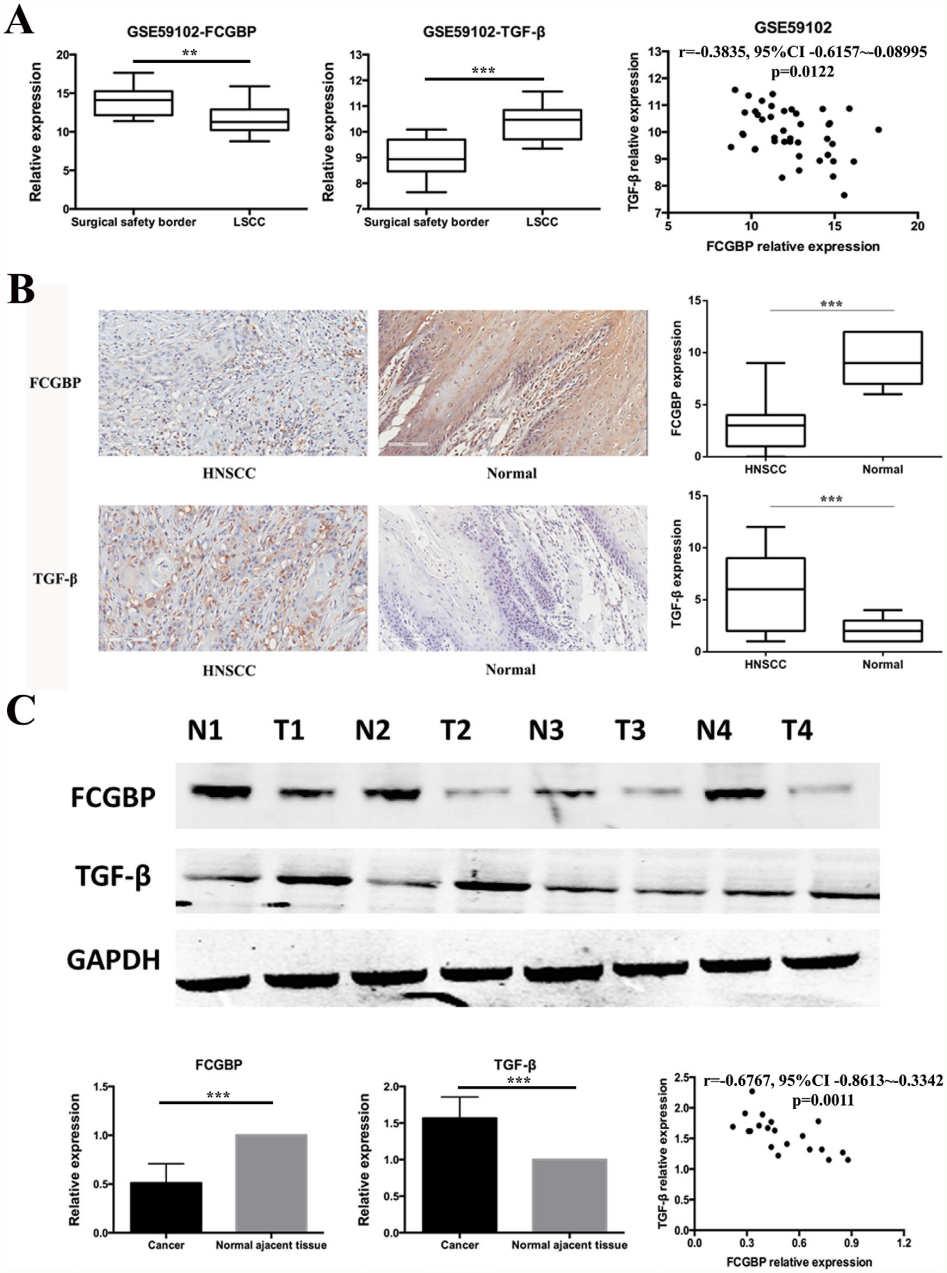
FcGBP and TGF-β expression in HNSCC and normal tissues **(A)** Data of GSE59102 showed that FcGBP mRNA expression level was lower in HNSCC compared to surgical safety border tissues (p=0.0015), while TGF-β mRNA expression level was higher in HNSCC compared to surgical safety border tissues (p<0.0001). mRNA expression level of FcGBP and TGF-β was negatively correlated in GSE59102 (r=-0.3835, 95%CI: -0.6157∼-0.08995, p=0.0122). **(B)** Results of IHC detection showed that FcGBP protein expression level was lower in HNSCC compared to surgical safety border tissues (p<0.0001), while TGF-β mRNA expression level was higher in HNSCC compared to surgical safety border tissues (p<0.0001). **(C)** Results of WB detection showed that FcGBP protein expression level was lower in HNSCC compared to surgical safety border tissues (p<0.0001), while TGF-β mRNA expression level was higher in HNSCC compared to surgical safety border tissues (p<0.0001). Protein expression level of FcGBP and TGF-β was negatively correlated in HNSCC and surgical safety border tissues (r=-0.6767, 95%CI: -0.8613∼-0.3342, p=0.0011). *p<0.05; **p<0.01; ***p<0.001.

First, we investigate FcGBP mRNA expression level in GEO dataset GSE59102, and we found that FcGBP was down regulated in HNSCC tissues compared to surgical safety border tissues (p=0.0015) (Figure 1A). Then we detect FcGBP protein expression level in 62 paired HNSCC and surgical safety border tissues, and we found that FcGBP protein expression level was lower in HNSCC compared to surgical safety border tissues (p<0.0001) (Figure 1C), and similar results generated in IHC experiment (p<0.0001) (Figure 1B). After that, we investigate TGF-β mRNA and protein expression levels in HNSCC and surgical safety border tissues, and we found that TGF-β mRNA and protein expression levels were both increased in HNSCC compared to surgical safety border (p<0.0001) (Figure 1A, 1B, 1C). Also, we found mRNA and protein expression level of FcGBP and TGF-β was significantly negative correlated in HNSCC and surgical safety border tissues (GSE59102: r=-0.3835, p=0.0122; WB: r=-0.6767, p=0.0011) (Figure 1A, 1C).

### FcGBP gene expression level was higher in HPV-positive HNSCC and correlated to longer OS time in HNSCC patients

**Figure 2:**
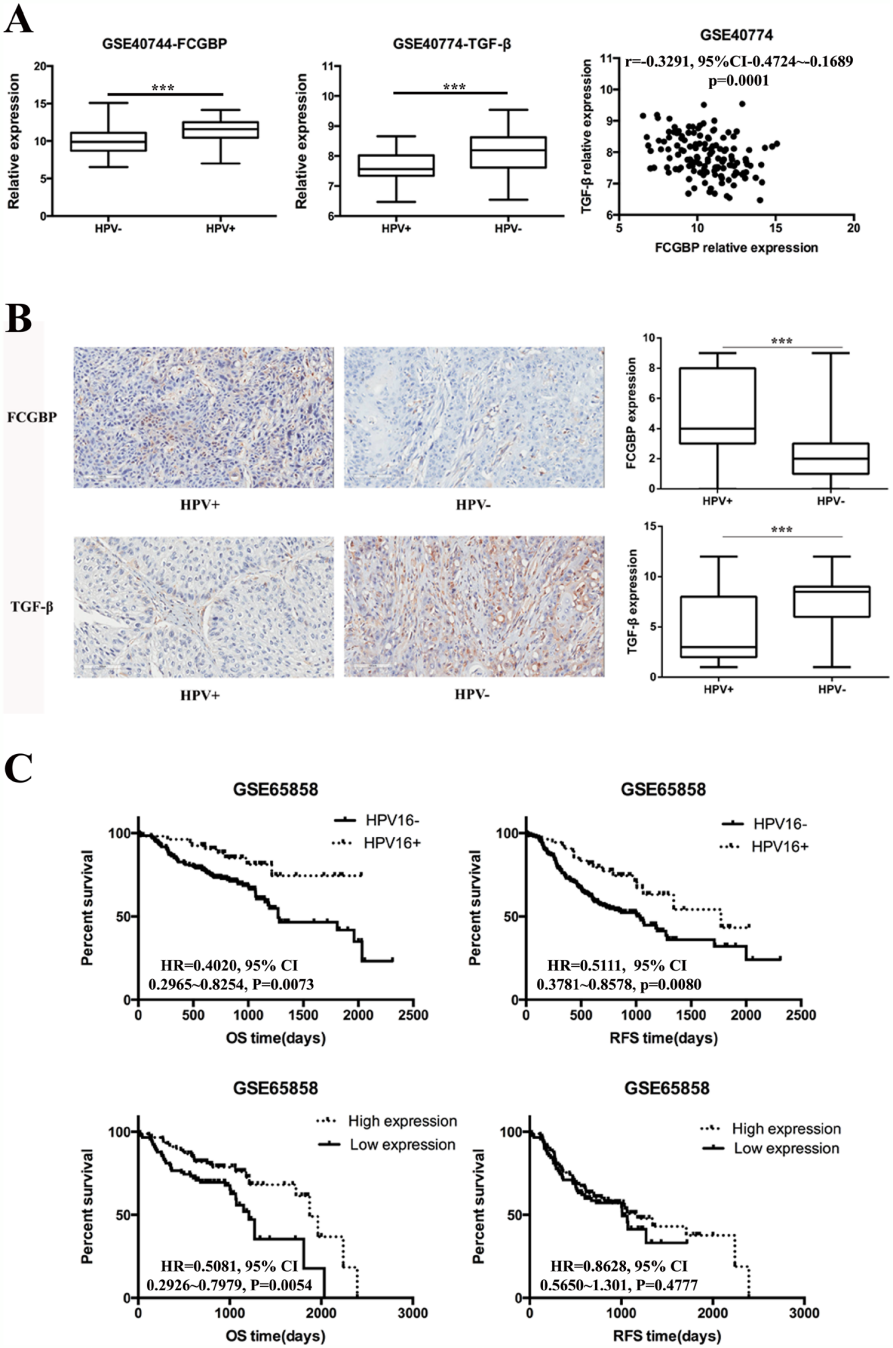
FcGBP and TGF-β expression in HPV-positive and HPV-negative HNSCC **(A)** Data of GSE40774 showed that FcGBP mRNA expression level was higher in HPV-positive HNSCC compared to HPV-negative HNSCC tissues (p<0.0001), while TGF-β mRNA expression level was lower in HPV-positive HNSCC compared to HPV-negative HNSCC tissues (p<0.0001). mRNA expression level of FcGBP and TGF-β was negatively correlated in GSE40774 (r=-0.3291, 95%CI: -0.4724∼-0.1689, p=0.0001). **(B)** Results of IHC detection showed that FcGBP protein expression level was higher in HPV-positive HNSCC compared to HPV-negative HNSCC tissues (p<0.0001), while TGF-β mRNA expression level was lower in HPV-positive HNSCC compared to HPV-negative HNSCC tissues (p<0.0001). **(C)** HPV-positive HNSCC patients had longer OS and RFS time than HPV-negative HNSCC patients (OS: HR=0.4020, 95% CI: 0.2965∼0.8254, p=0.0073; RFS: HR=0.5111, 95% CI:0.3781∼0.8578, p=0.0080). Higher FcGBP mRNA expression level was significantly correlated with longer OS but not RFS time in patients with HNSCC (OS: HR=0.5081, 95% CI: 0.2926∼0.7979, P=0.0054; RFS: HR=0.8628, 95% CI: 0.5650∼1.301, P=0.4777). *p<0.05; **p<0.01; ***p<0.001.

After we determined the expression level of FcGBP and TGF-β in HNSCC and surgical safety border tissues, we pay attention to verify the gene expression difference of FcGBP and TGF-β in HPV-positive and HPV-negative HNSCC tissues. We found FcGBP mRNA expression level was higher in HPV-positive HNSCC tissues compared to HPV-negative HNSCC tissues in GSE40774 (p<0.0001), while TGF-β mRNA expression level was lower in HPV-positive HNSCC tissues compared to HPV-negative HNSCC tissues in GSE40774 (p<0.0001) (Figure 2A). Next we detect FcGBP and TGF-β protein expression level in HPV-positive and HPV-negative HNSCC tissues. Similar to GEO dataset analysis, FcGBP protein expression level was higher in HPV-positive HNSCC tissues (p<0.001), and TGF-β protein expression level was lower in HPV-positive HNSCC tissues (p<0.001) (Figure 2B). Also, FcGBP and TGF-β mRNA expression level was negative correlated in HPV-positive and HPV-negative HNSCC tissues (GSE40774: r=-0.3291, p=0.0001) (Figure 2A).

We further investigated the role of HPV status and FcGBP expression level in the prognosis of patients with HNSCC. The mRNA expression data from GSE65858 showed that FCGBP mRNA expression level and HPV status were highly correlated with OS and RFS time in patients with HNSCC. HPV-negative HNSCC patients had shorter OS and RFS time than HPV-positive HNSCC patients (OS: HR=0.3750, p=0.0157; RFS: HR=0.4434, p=0.0080) (Figure 2C). Meanwhile, HNSCC patients with higher expression level of FcGBP mRNA had longer OS time than HNSCC patients with lower FcGBP mRNA expression level (OS: HR=0.5081, p=0.0054) (Figure 2C).

### FcGBP overexpression and exogenous TGF-β treatment could modulate proliferation, migration capability in HNSCC cells

**Figure 3:**
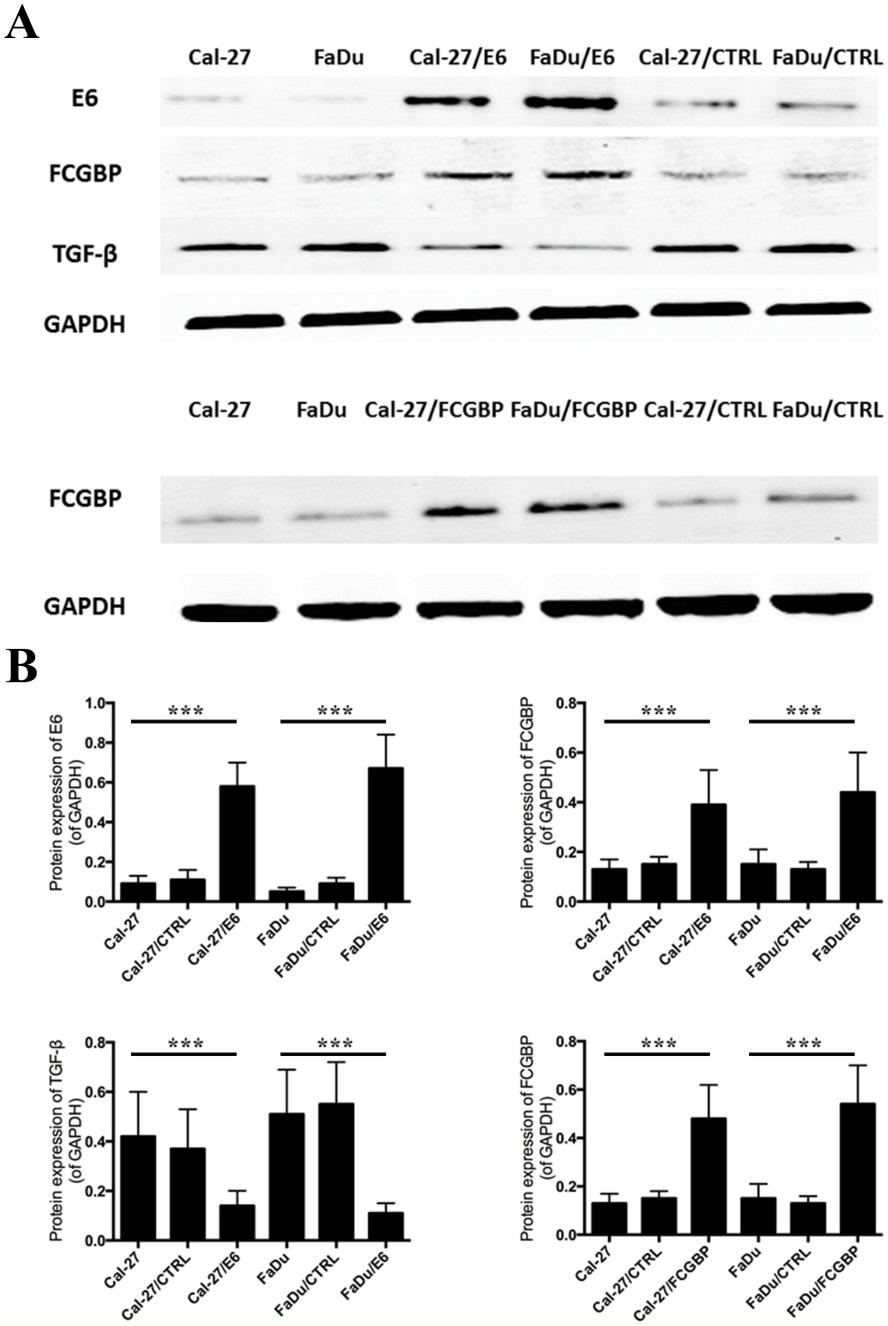
FcGBP and TGF-β protein expression after HPV E6 and FcGBP overexpression in HNSCC Cal-27 and FaDu cells **(A)** Results of WB detection showed that FcGBP protein expression level increased after HPV E6 overexpression in HPV-negative HNSCC Cal-27 and FaDu cells, while TGF-β protein expression level decreased after HPV E6 overexpression. **(B)** Represent histograms of (A) showed the difference of FcGBP and TGF-β protein expression level after HPV E6 and FcGBP overexpression in HNSCC Cal-27 and FaDu cells. *p<0.05; **p<0.01; ***p<0.001.

**Figure 4:**
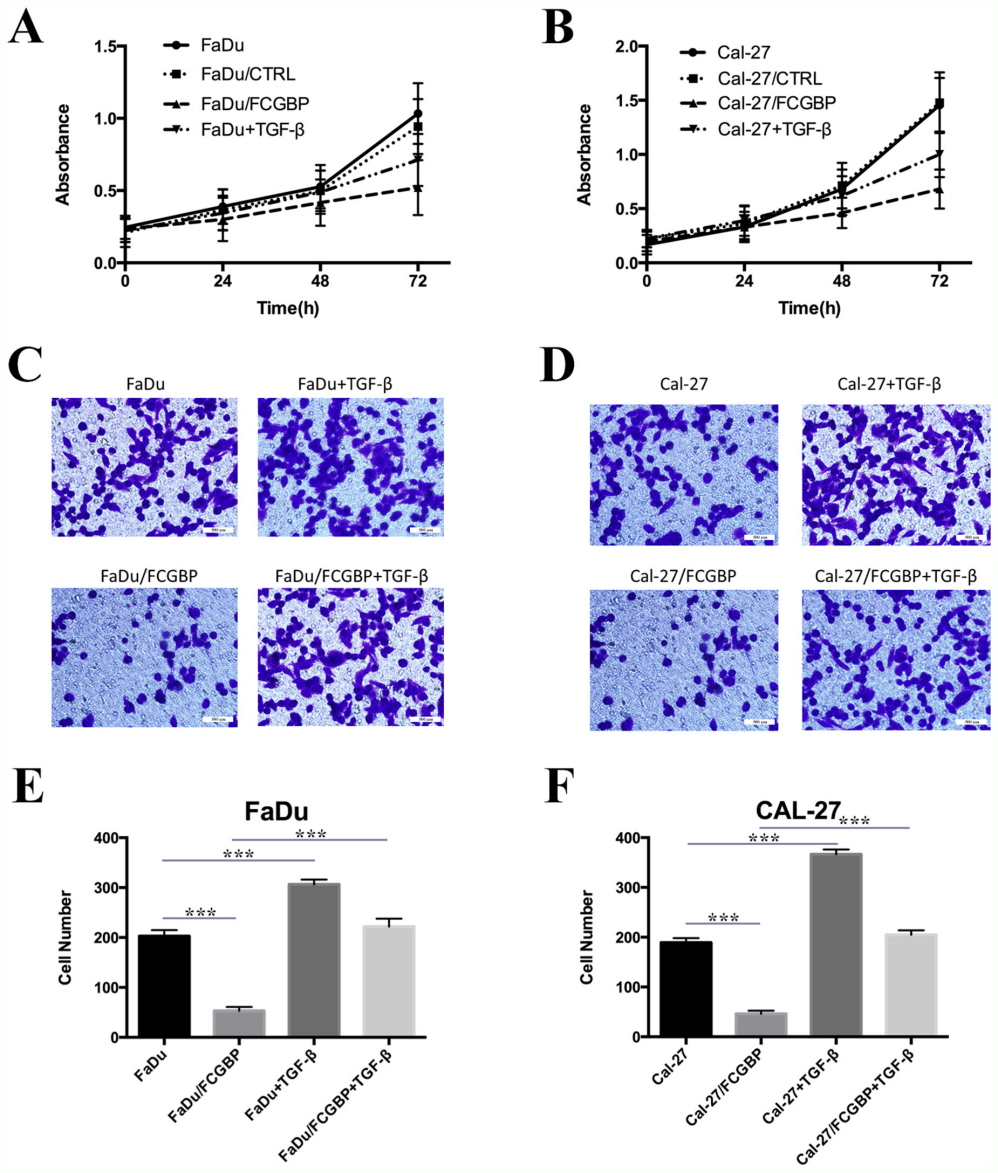
Proliferation and migration capacity change of HNSCC Cal-27 and FaDu cells after FcGBP overexpression **(A)** Multiplication curve of FaDu, FaDu/CTRL, FaDu/FcGBP and FaDu/FcGBP+ 5ng/ml TGF-β. **(B)** Multiplication curve of Cal-27, Cal-27/CTRL, Cal-27/FcGBP and Cal-27/FcGBP+ 5ng/ml TGF-β. **(C)** Transwell detection results of FaDu, FaDu/CTRL, FaDu/FcGBP and FaDu/FcGBP+ 5ng/ml TGF-β. **(D)** Transwell detection results of Cal-27, Cal-27/CTRL, Cal-27/FcGBP and Cal-27/FcGBP+ 5ng/ml TGF-β. **(E)** Represent histogram of (C) showed the difference of cell numbers between four groups. **(F)** Represent histogram of (D) showed the difference of cell numbers between four groups. *p<0.05; **p<0.01; ***p<0.001.

Since we identify that FcGBP gene expression decreased in HNSCC and correlated with poor prognosis in patients with HNSCC, we ectopic overexpress FcGBP in HNSCC FaDu and Cal-27 cells (Figure 3). We found that proliferation rate of FaDu and Cal-27 cells both significantly decreased after FcGBP overexpression, and the inhibition rate of proliferation of FaDu and Cal-27 was 49.6% and 53.2% at 72h. Meanwhile, proliferation rate of FaDu and Cal-27 cells both decreased after 5ng/ml TGF-β treatment, and the inhibition rate of proliferation of FaDu and Cal-27 was 30.1% and 31.2% at 72h (Figure 4A, 4B).

Results of transwell test showed that FcGBP overexpression could inhibit the migration capability of FaDu and Cal-27 cells, and the cell numbers migrated through the membrane were 53±8 and 46±7 at 48h, compared to control group (FaDu: 203±12 and Cal-27: 189±9). TGF-β could enhance the migration capability of FaDu and Cal-27 cells, and TGF-β could enhance the migration capability of FaDu/FcGBP and Cal-27/FcGBP too (Figure 4C, 4D, 4E, 4F).

### HPV E6 overexpression and exogenous TGF-β modulated FcGBP gene expression in HNSCC cells

Since we found FcGBP gene expression was significantly different between HPV-positive and HPV-negative HNSCC tissues. We overexpressed HPV E6 in HPV-negative HNSCC FaDu and Cal-27 cells, and we found FcGBP gene expression level increased after HPV E6 overexpression along with TGF-β gene expression level decreased (Figure 3).

**Figure 5:**
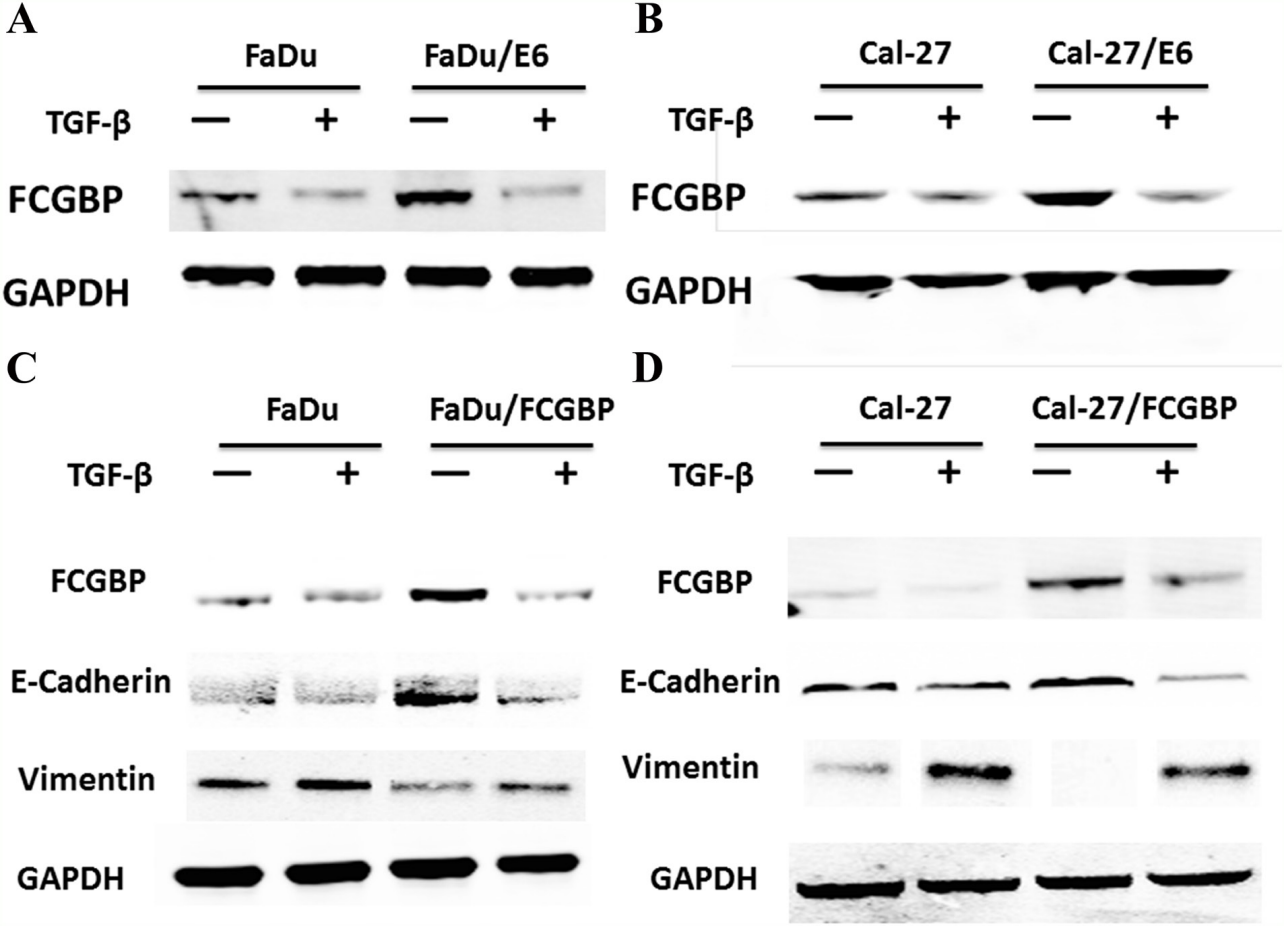
Protein expression level of FcGBP and EMT markers in HNSCC FaDu and Cal-27 cells after HPV E6 and FcGBP overexpression **(A)** FcGBP protein expression level in FaDu and FaDu/E6 along with 5ng/ml TGF-β treatment or not. **(B)** FcGBP protein expression level in Cal-27 and Cal-27/E6 along with 5ng/ml TGF-β treatment or not. **(C)** Protein expression level of FcGBP and EMT markers in FaDu and FaDu/FcGBP along with 5ng/ml TGF-β treatment or not. **(D)** Protein expression level of FcGBP and EMT markers in Cal-27 and Cal-27/FCGBP along with 5ng/ml TGF-β treatment or not.

We found that FcGBP gene expression level decreased in FaDu and Cal-27 cells after 5ng/ml TGF-β treatment for 48h. Next, we investigated FcGBP gene expression level in FaDu/FcGBP and Cal-27/FcGBP cells after 5ng/ml TGF-β treatment for 48h, and we found the same results (Figure 5A, 5B).

**Figure 6:**
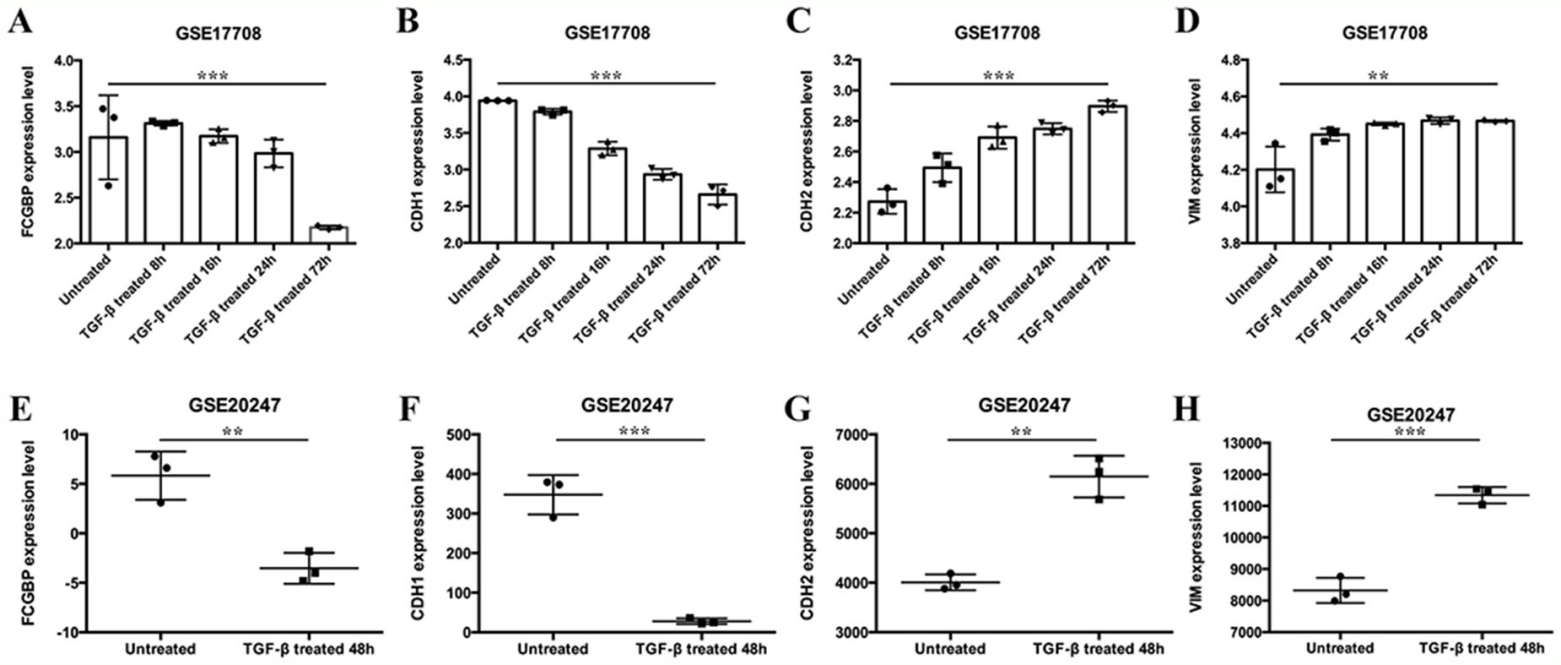
mRNA expression level of FCGBP and EMT markers after TGF-β treatment Data of GSE 17708 showed that **(A)** FcGBP, **(B)** CDH1, **(C)** CDH2, **(D)** VIM mRNA expression level after TGF-β treatment for 8h, 16h, 24h and 72h in lung adenocarcinoma A549 cells. Data of GSE20247 showed that **(E)** FcGBP, **(F)** CDH1, **(G)** CDH2, **(H)** VIM mRNA expression level after TGF-β treatment for 48h in human proximal tubular HK-2 cells. *p<0.05; **p<0.01; ***p<0.001.

Then we analyzed data from GSE17708 and GSE20247, and we found that FcGBP mRNA expression level was decreased after exogenous TGF-β treatment (p=0.0006; p=0.0050, respectively) (Figure 6A, 6E).

### FcGBP gene expression correlated with EMT

We found the migration capability of HNSCC FaDu and Cal-27 cells was inhibited after FcGBP overexpression. Furthermore, we found that EMT was inhibited after FcGBP overexpression in HNSCC FaDu and Cal-27 cells, E-Cadherin (CDH1) expression level increased while Vimentin expression decreased. TGF-β could inhibit FcGBP gene expression and activate EMT in FaDu, Cal-27 and FaDu/FcGBP, Cal-27/FcGBP cells (Figure 5C, 5D).

Also, we found that FcGBP gene expression level was negatively correlated to Vimentin and N-Cadherin (CDH2) gene expression level while positively correlated with CDH1 gene expression level in GSE17708 and GSE20247. Meanwhile, data from GSE17708 and GSE20247 showed that CDH2 and Vimentin expression level increased while CDH1 expression level decreased in consequence of EMT activation after exogenous TGF-β treatment (Figure 6B, 6C, 6D, 6F, 6G, 6H).

## DISCUSSION

Head and neck squamous cell carcinoma (HNSCC) accounts for ∼6% of all human cancers, with ∼650,000 new cases reported and 350,000 HNSCC-related deaths per year worldwide. Although the treatment has been technically and medically improved during the last decades, the 5-year overall survival (OS) rate is still stagnating at about 50% [18], local recurrence and metastasis were the main cause of death in HNSCC patients. Meanwhile, the treatment effect of many other types of tumors has been improved, the patient's survival time is prolonged, and the quality of living is improved [19–21].

HPV infection is an important etiology of HNSCC, but HPV-positive and HPV-negative HNSCC have different biological behavior and clinical outcomes due to HPV infection. HPV-positive HNSCC patients have a significantly better prognosis than HPV-negative patients, with the 3- and 5-year survival at 84% and 62% for HPV-positive patients compared to 57% and 26% for HPV-negative patients, respectively [7]. In present study, we referenced the results of GSE65858, and found that HPV-positive correlated with longer OS and RFS time compared to HPV-negative HNSCC patients.

FcGBP was first identified as an Fc portion of the IgG molecule binding site in intestinal and colonic epithelia, gallbladder, cystic duct, bronchus, submandibular gland, cervix uteri and in fluids secreted by these cells in humans. It might play a role in cell protection and anti-inflammation in tissues [15]. FcGBP gene expression is significantly decreased in colon cancer carcinogenesis [22]. Griffith et al. showed that FcGBP was differentially expressed in normal thyroid tissue, thyroid adenomas and thyroid carcinomas [23]. We found FcGBP gene expression was down regulated in HNSCC tissues, and correlated to OS time of HNSCC patients. The proliferation and migration capability was inhibited after FcGBP over-expression in HNSCC FaDu and Cal-27 cells.

FcGBP is composed of many repeated domains, such as thirteen Von Willebrand factor D domains, and twelve cysteine rich (Cys-rich) and twelve trypsin inhibitor-like domains. The Cys-rich domains are essential in the formation of many disulfide bridges with another molecules, plausibly resulting in a net-like scaffold within the mucosal barrier [12]. In the intestine, FcGBP protein exists in a large complex containing Muc2 and Trefoil peptide [24, 25]. These results suggest that it may be important in the maintenance of homeostasis and integrity of epithelium.

Meanwhile, we found that FcGBP was lower in HPV-negative HNSCC tissues compared to HPV-positive HNSCC tissues. We assume that FcGBP gene expression could be modulated by HPV infection. FcGBP gene located in 19q 13.2, and genome research found that gain of 19q chromosome occurs in about 23.1% HPV-positive HNSCC patients [26], and this might explain why FcGBP gene expression level was higher in HPV-positive HNSCC patients.

In present investigation, we found that both mRNA and protein expression level of FcGBP was negative correlated with TGF-β in HNSCC and surgical safety border tissues. Currently TGF-β is understood to act as a tumor-suppressor early in tumorigenesis, and then in later phases it enhances the malignant phenotype. Thus, the expression levels of TGF-β are usually reduced during tumorigenesis, and the expression of TGF-β is increased during malignant progression [27, 28]. TGF-β activities bifurcate to exert anti-proliferative and pro-EMT actions in HPV-transformed cells [29]. In present investigation, we found that TGF-β expression level was higher in HNSCC tissues than in normal tissues along with another reports, also we found that TGF-β gene expression was higher in HPV-negative HNSCC patients compared to HPV-positive HNSCC patients. Małgorzata et al. found that TGF-β level was lower in saliva of patients with HPV infection [30]. We found TGF-β gene expression was down regulated after HPV E6 transfection in HPV-negative HNSCC FaDu and Cal-27 cells. These results indicated that TGF-β gene expression was inhibited by HPV infection in HNSCC patients. We found TGF-β could inhibit FcGBP gene expression *in vitro*, and the results of GSE17708 and GSE20247 confirmed these findings.

EMT is a key incident in HNSCC development, and TGF-β is a primary factor triggering EMT in HNSCC [31]. In present investigation, we found that EMT could be activated by exogenous TGF-β treatment, and EMT could be inhibited by FcGBP over-expression in HNSCC cells. Exogenous TGF-β could counteract the EMT inhibition caused by FcGBP overexpression.

**Figure 7:**
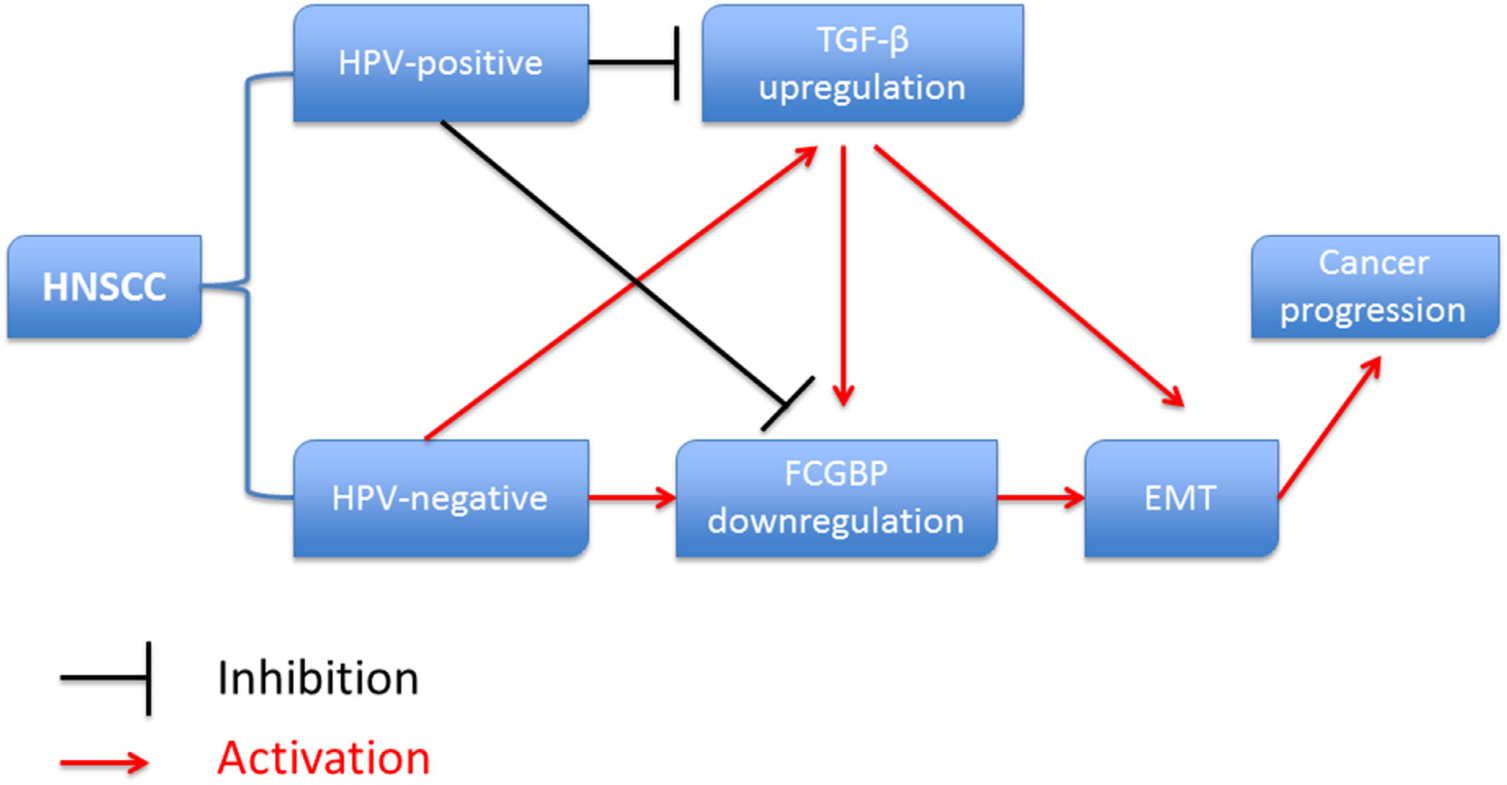
Diagram showed the relationship between HPV infection, TGF-β, FcGBP and EMT in HNSCC development

In conclusion, FcGBP gene expression was decreased in HNSCC, and lower FcGBP expression was correlated with shorter OS time of HNSCC patients. HPV-positive was a good prognostic factor in HNSCC patients, and TGF-β expression was inhibited by HPV infection. FcGBP gene expression was modulated by HPV infection. TGF-β mediated EMT activation played an important role in this progress (Figure 7).

## MATERIALS AND METHODS

### Cell culture and main reagents

The human HPV-negative HNSCC cell lines, FaDu and Cal-27, were cultured in RMPI1640 with 10% FBS at 37°C, 5% CO_2_. Fetal bovine serum (FBS) was obtained from Gibco (Logan, UT, USA). RPMI-1640 media and 0.25% trypsin solution were purchased from Invitrogen (Carlsbad, USA). Recombinant TGF-β protein was obtained from RD (Minneapolis, MN, USA). Cells in logarithmic growth phase were used for experiment; FcGBP, HPV E6 and TGF-β anti-body were purchased from Sigma-Aldrich (St. Louis, USA); E-Cadherin, Vimentin and GAPDH anti-body were purchased from Cell Signaling technology (Danvers, MA, USA). 10% Bis-Tris pLUS GELs was obtain from Thermo-fisher Technology (Waltham, MA USA).

### HNSCC associated database obtained from GEO database

In this study, 5 HNSCC associated datasets, GSE17708, GSE20247, GSE40774, GSE59102 and GSE65858, were downloaded from GEO database, respectively. GABRP mRNA expression data was extracted and analyzed. Detailed information could be found in Table 1.

**Table 1:** Detailed information of GEO datasets selected

GEO number	Title	Platform	Sample number
GSE17708	Time Course of TGF-beta treatment of A549 lung adenocarcinoma cell line	GPL570	26
GSE20247	C-peptide and/or transforming growth factor beta 1 effect on human proximal tubular cell line	GPL6884	18
GSE40774	Integrative genomic analysis identifies clinically relevant subtypes of head and neck cancer characterized by hypoxia, T-cell infiltration, and EMT	GPL13497	134
GSE59102	HOX genes: potential candidates furthering the development of larynx squamous cell carcinoma	GPL6480	42
GSE65858	Gene expression patterns and TP53 mutations are associated with HPV RNA status, lymph node metastasis, and survival in head and neck cancer	GPL10558	270

### HNSCC tissue samples

62 patients were selected to enter the study (Table 2). The diagnosis and HPV status were confirmed based on clinical, and histopathological or cytological examination. This study was approved by the ethics committee of The Sixth Affiliated Hospital, Wenzhou medical University, and all specimens were collected from patients provided written informed consent in accordance with the principles of the Declaration of Helsinki and Good Clinical Practice Guidelines.

**Table 2:** Clinical and pathological information of HNSCC patients

Characteristic	Number (N=62)	P value
**Sex**		P<0.01
Male	46	
Female	16	
**Age**		P<0.01
≥45	50	
<45	12	
**Tumor location**		P>0.05
Glottis	24	
Supraglottis	10	
Hypopharynx	18	
Nasopharynx	10	
**Differentiation grade**		P>0.05
Poor	20	
Moderate	14	
Well	28	
**HPV status**		P<0.01
HPV-positive	13	
HPV-negative	49	

### Immunohistochemical staining and evaluation of results

IHC staining was performed as described previously [32]. The primary mouse anti-FcGBP antibody (1:100) was used. For IHC staining evaluation, immunoreactivity was assessed by two blinded independent observers using light microscopy (Olympus BX-41 light microscope). FCGBP protein intensity and frequency were transformed into a Composit Expression Score (CES) utilizing the formula CES=Intensity× Frequency. The range of CES was from 0 to 12. The CES was scored as negative (0), weak positive (1∼4), positive (5∼8), strong positive (9∼12).

### Gene transfection

HPV E6 cDNA was cloned into plasmid vector pcDNA3.1-EGFP. After amplification and DNA sequence confirmation, this plasmid was used to overexpress HPV E6 in HPV-negative HNSCC FaDu and Cal-27 cells. Briefly, Cells were grown and stably transfected with plasmid-E6 or plasmid-empty for control. Cells were treated with 400μg/ml neomycin/G418 for 14d after transfection, and named FaDu/E6, Cal-27/E6 or FaDu/CTRL, Cal-27/CTRL.

We use adenovirus-based FcGBP vector to overexpress FcGBP gene expression in FaDu and Cal-27 cells. Briefly, cells were grown and transfected with adenovirus-based FcGBP vector and empty adenovirus vector. Then, cells were treated with 1ng/ml puromycin for 14 days after transfection, and named FaDu/FcGBP, Cal-27/FcGBP or FaDu/CTRL, Cal-27/CTRL.

### Cell proliferation test

We use Cell Counting Kit-8 (CCK-8) to investigate the proliferation of HNSCC cell lines. Cells at logarithmic phase were seeded in 96 well plates with 1×10^3^ cells per well for normal culture. Six double wells were set in every group, and a blank control was also used. After 24 h, 48 h and 72 h culture, 10μl of CCK-8 regent was added in each well, and incubated at 37°C for 1 hour, and absorbance value was measured at 450 nm.

### Cell migration and invasion test

Cell migration and invasion assays were performed using Transwell migration chambers according to the manufacturer's protocol. In brief, 1x10^4^ cells were suspended in 200 μl serum-free RPMI-1640 medium. The cells were seeded in the upper chamber; the lower chamber contained RPMI-1640 medium with 10% FBS as the chemoattractant. After incubation for 48 h at 37°C in a humidified atmosphere of 5% CO2, any cells that had not penetrated the membrane were removed using cotton swabs; the cells that had successfully migrated to the bottom surfaces of the membranes were fixed with 4% polyoxymethylene and stained with 0.1% crystal violet for 20 min. They were counted under a microscope.

### Western blotting

Total cell protein extracts were prepared following the manufacturer's instructions. The lysates were resolved using 10% Bis-tris GEL, transferred to PVDF membranes and immunoblotted with primary antibodies against HPV E6, FcGBP, TGF-β, E-Cadherin, Vimentin and GAPDH. Following incubation with secondary antibodies, the protein bands were detected using an enhanced chemiluminescence reagent (Thermo Fisher Scientific, Rockford, IL, USA).

### The statistical analysis

All experiments were performed three times. All statistical analyses were performed using the SPSS 16.0 software. Quantitative data was expressed as means ± SD. Student's t-test was used to compare the differences of means between each two groups. One-way ANOVA was used to analyze the differences between multiple sets of data Pearson Correlation Coefficient analyzed correlation between two groups. The Kaplan-Meier method, Hazard ratios (HR), 95% confidence intervals (CI) and log-rank test were used to evaluate the correlation between FcGBP expression and patient survival. The criterion for statistical significance was established at P<0.05.
